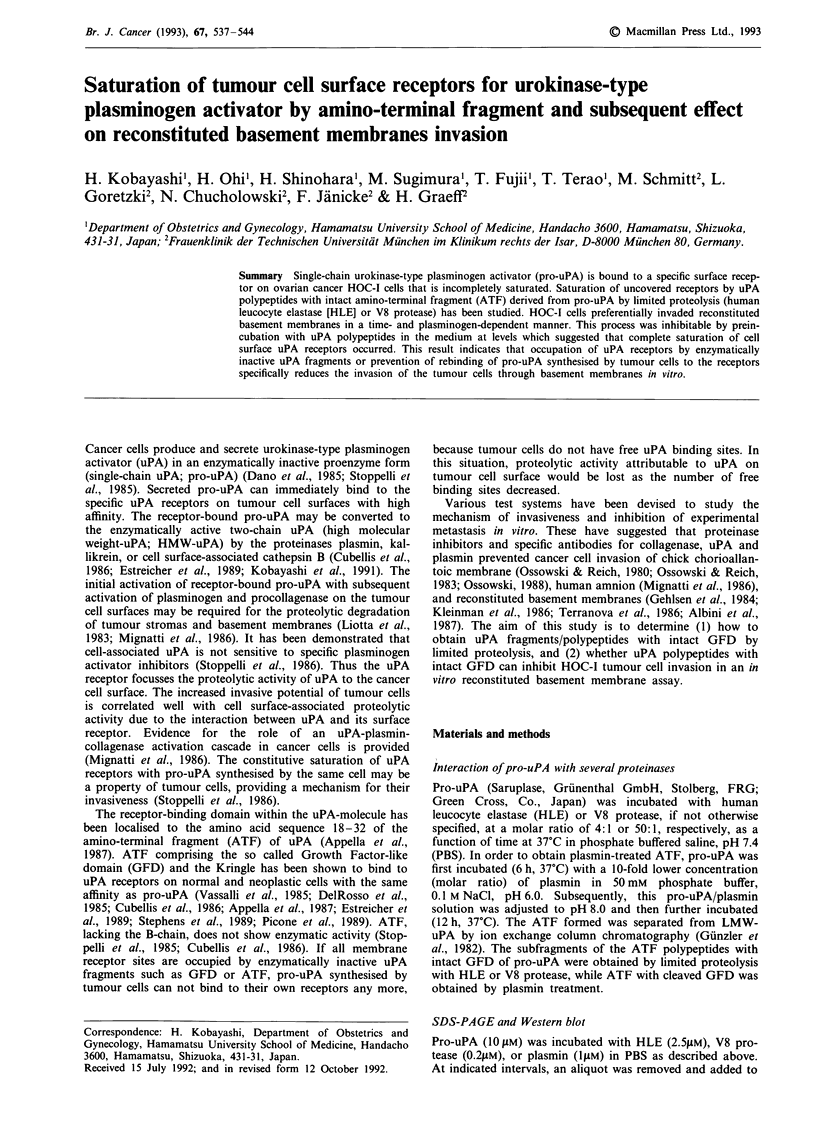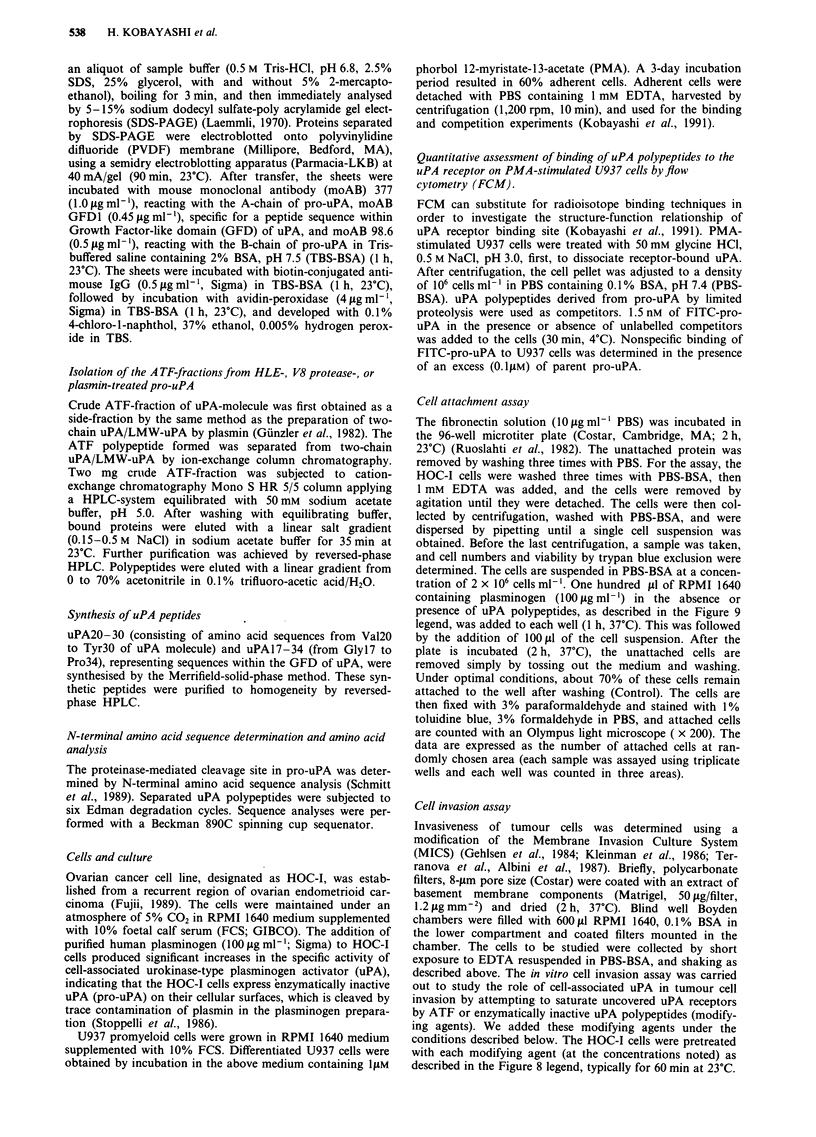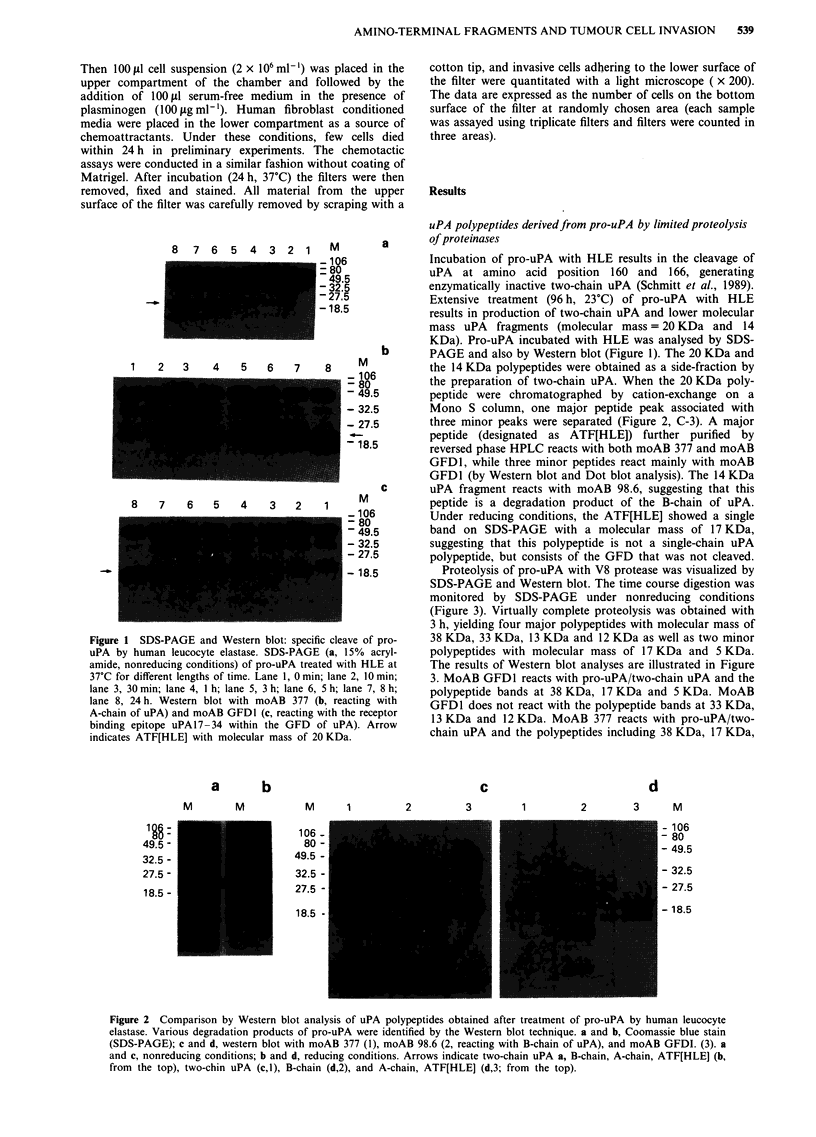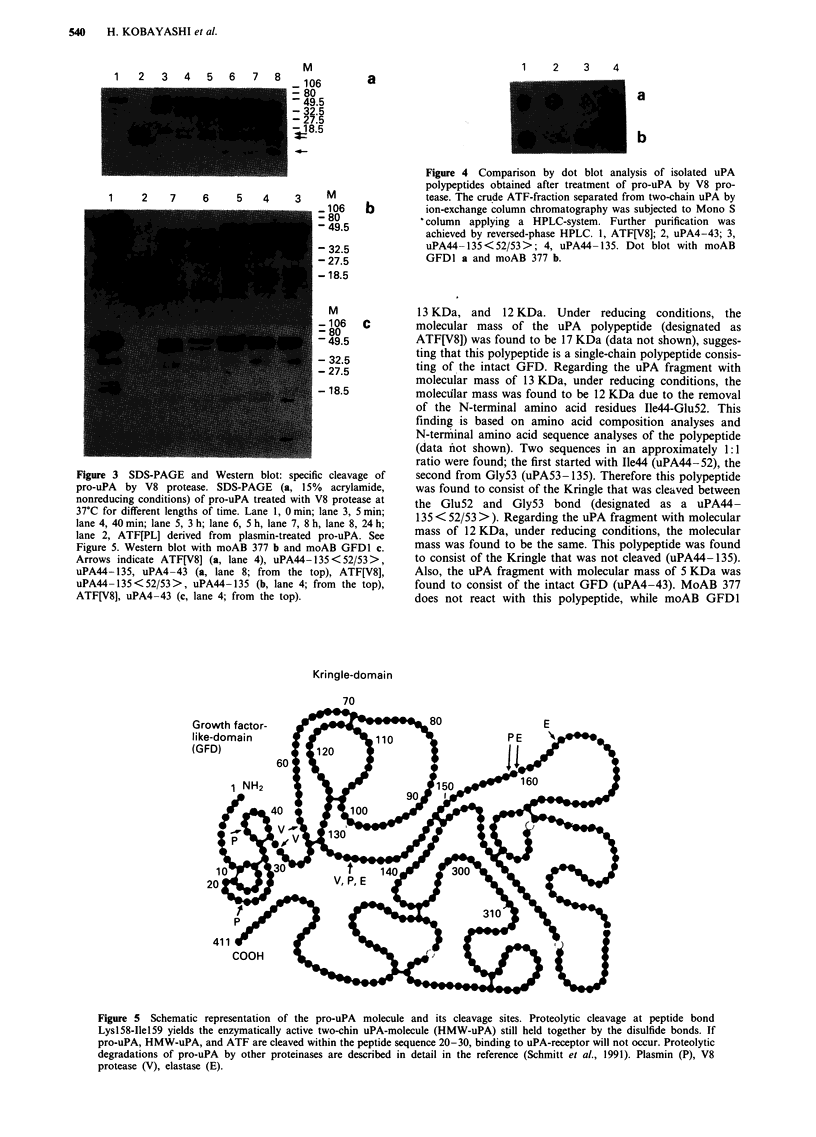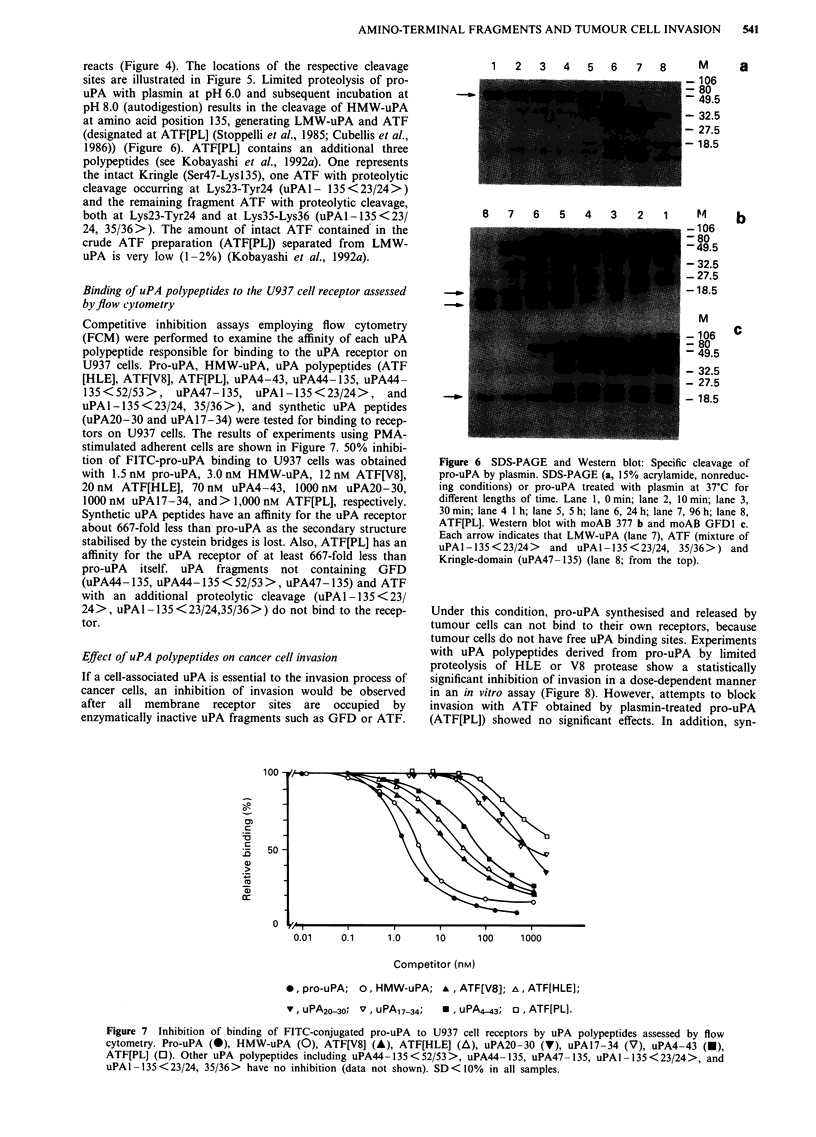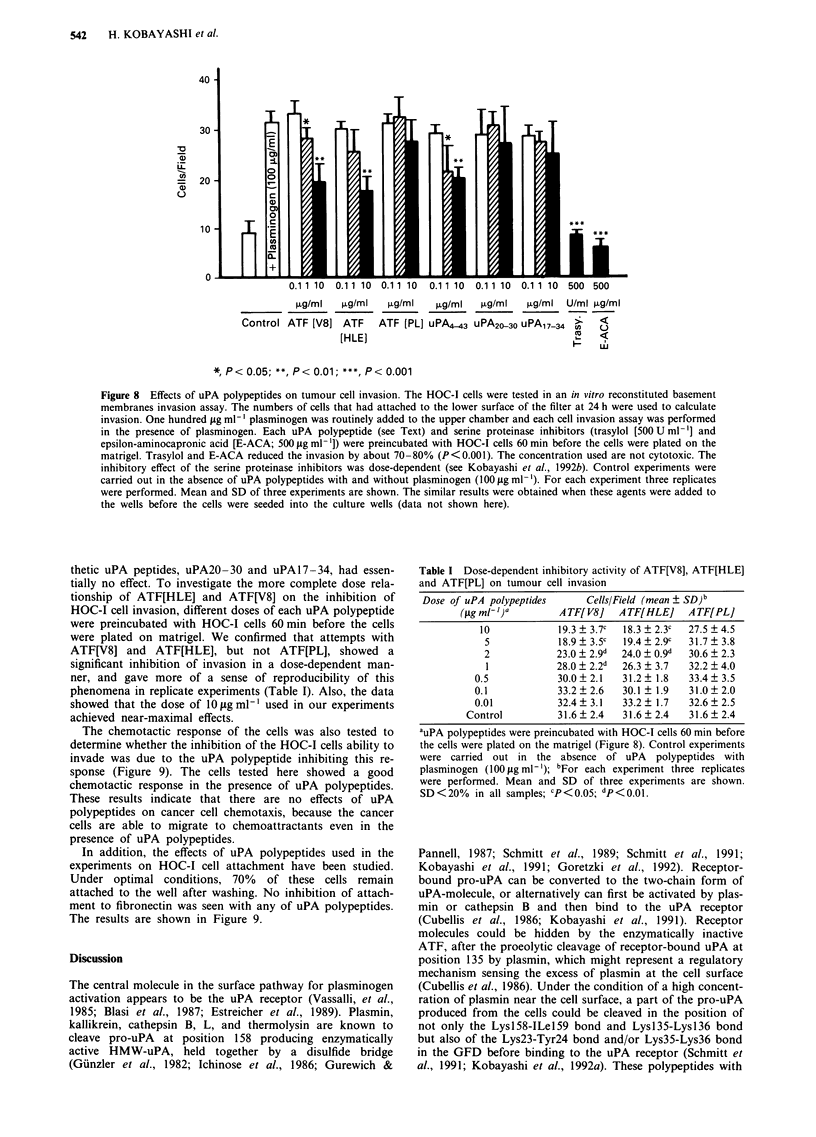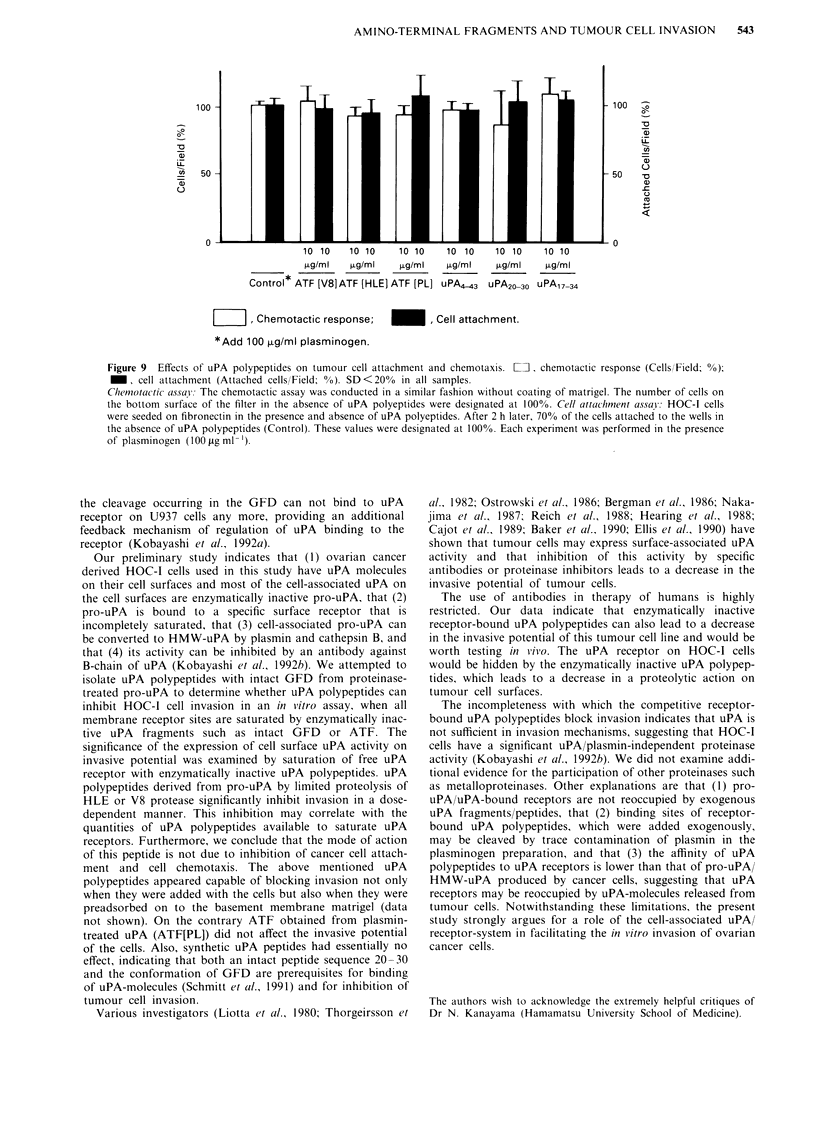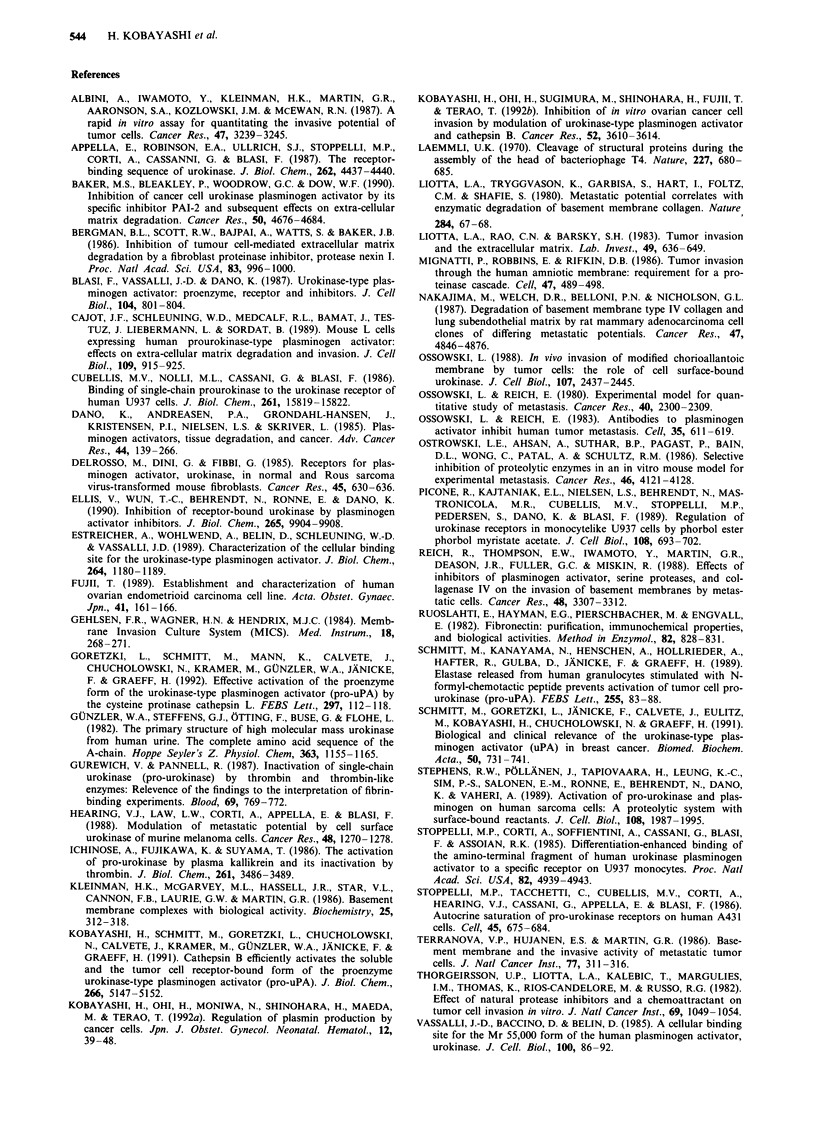# Saturation of tumour cell surface receptors for urokinase-type plasminogen activator by amino-terminal fragment and subsequent effect on reconstituted basement membranes invasion.

**DOI:** 10.1038/bjc.1993.99

**Published:** 1993-03

**Authors:** H. Kobayashi, H. Ohi, H. Shinohara, M. Sugimura, T. Fujii, T. Terao, M. Schmitt, L. Goretzki, N. Chucholowski, F. Jänicke

**Affiliations:** Department of Obstetrics and Gynecology, Hamamatsu University School of Medicine, Shizuoka, Japan.

## Abstract

**Images:**


Br. J. Cancer (1993), 67, 537–544 © Macmillan Press Ltd., 1993

### Saturation of tumour cell surface receptors for urokinase-type plasminogen activator by amino-terminal fragment and subsequent effect on reconstituted basement membranes invasion

H. Kobayashi[1], H. Ohi[1], H. Shinohara[1], M. Sugimura[1], T. Fujii[1], T. Terao[1], M. Schmitt[2], L. Goretzki[2], N. Chucholowski[2], F. Jänicke[2] & H. Graeff[2]

[1]*Department of Obstetrics and Gynecology, Hamamatsu University School of Medicine, Handacho 3600, Hamamatsu, Shizuoka, 431-31, Japan;* [2]*Frauenklinik der Technischen Universität München im Klinikum rechts der Isar, D-8000 München 80, Germany.*

**Summary** Single-chain urokinase-type plasminogen activator (pro-uPA) is bound to a specific surface receptor on ovarian cancer HOC-I cells that is incompletely saturated. Saturation of uncovered receptors by uPA polypeptides with intact amino-terminal fragment (ATF) derived from pro-uPA by limited proteolysis (human leucocyte elastase [HLE] or V8 protease) has been studied. HOC-I cells preferentially invaded reconstituted basement membranes in a time- and plasminogen-dependent manner. This process was inhibitable by preincubation with uPA polypeptides in the medium at levels which suggested that complete saturation of cell surface uPA receptors occurred. This result indicates that occupation of uPA receptors by enzymatically inactive uPA fragments or prevention of rebinding of pro-uPA synthesised by tumour cells to the receptors specifically reduces the invasion of the tumour cells through basement membranes *in vitro*.

Cancer cells produce and secrete urokinase-type plasminogen activator (uPA) in an enzymatically inactive proenzyme form (single-chain uPA; pro-uPA) (Dano *et al.*, 1985; Stoppelli *et al.*, 1985). Secreted pro-uPA can immediately bind to the specific uPA receptors on tumour cell surfaces with high affinity. The receptor-bound pro-uPA may be converted to the enzymatically active two-chain uPA (high molecular weight-uPA; HMW-uPA) by the proteinases plasmin, kallikrein, or cell surface-associated cathepsin B (Cubellis *et al.*, 1986; Estreicher *et al.*, 1989; Kobayashi *et al.*, 1991). The initial activation of receptor-bound pro-uPA with subsequent activation of plasminogen and procollagenase on the tumour cell surfaces may be required for the proteolytic degradation of tumour stromas and basement membranes (Liotta *et al.*, 1983; Mignatti *et al.*, 1986). It has been demonstrated that cell-associated uPA is not sensitive to specific plasminogen activator inhibitors (Stoppelli *et al.*, 1986). Thus the uPA receptor focusses the proteolytic activity of uPA to the cancer cell surface. The increased invasive potential of tumour cells is correlated well with cell surface-associated proteolytic activity due to the interaction between uPA and its surface receptor. Evidence for the role of an uPA-plasmin-collagenase activation cascade in cancer cells is provided (Mignatti *et al.*, 1986). The constitutive saturation of uPA receptors with pro-uPA synthesised by the same cell may be a property of tumour cells, providing a mechanism for their invasiveness (Stoppelli *et al.*, 1986).

The receptor-binding domain within the uPA-molecule has been localised to the amino acid sequence 18–32 of the amino-terminal fragment (ATF) of uPA (Appella *et al.*, 1987). ATF comprising the so called Growth Factor-like domain (GFD) and the Kringle has been shown to bind to uPA receptors on normal and neoplastic cells with the same affinity as pro-uPA (Vassalli *et al.*, 1985; DelRosso *et al.*, 1985; Cubellis *et al.*, 1986; Appella *et al.*, 1987; Estreicher *et al.*, 1989; Stephens *et al.*, 1989; Picone *et al.*, 1989). ATF, lacking the B-chain, does not show enzymatic activity (Stoppelli *et al.*, 1985; Cubellis *et al.*, 1986). If all membrane receptor sites are occupied by enzymatically inactive uPA fragments such as GFD or ATF, pro-uPA synthesised by tumour cells can not bind to their own receptors any more, because tumour cells do not have free uPA binding sites. In this situation, proteolytic activity attributable to uPA on tumour cell surface would be lost as the number of free binding sites decreased.

Various test systems have been devised to study the mechanism of invasiveness and inhibition of experimental metastasis *in vitro*. These have suggested that proteinase inhibitors and specific antibodies for collagenase, uPA and plasmin prevented cancer cell invasion of chick chorioallantoic membrane (Ossowski & Reich, 1980; Ossowski & Reich, 1983; Ossowski, 1988), human amnion (Mignatti *et al.*, 1986), and reconstituted basement membranes (Gehlsen *et al.*, 1984; Kleinman *et al.*, 1986; Terranova *et al.*, 1986; Albini *et al.*, 1987). The aim of this study is to determine (1) how to obtain uPA fragments/polypeptides with intact GFD by limited proteolysis, and (2) whether uPA polypeptides with intact GFD can inhibit HOC-I tumour cell invasion in an *in vitro* reconstituted basement membrane assay.

### Materials and methods

### *Interaction of pro-uPA with several proteinases*

Pro-uPA (Saruplase, Grünenthal GmbH, Stolberg, FRG; Green Cross, Co., Japan) was incubated with human leucocyte elastase (HLE) or V8 protease, if not otherwise specified, at a molar ratio of 4:1 or 50:1, respectively, as a function of time at 37°C in phosphate buffered saline, pH 7.4 (PBS). In order to obtain plasmin-treated ATF, pro-uPA was first incubated (6 h, 37°C) with a 10-fold lower concentration (molar ratio) of plasmin in 50 mM phosphate buffer, 0.1 M NaCl, pH 6.0. Subsequently, this pro-uPA/plasmin solution was adjusted to pH 8.0 and then further incubated (12 h, 37°C). The ATF formed was separated from LMW-uPA by ion exchange column chromatography (Günzler *et al.*, 1982). The subfragments of the ATF polypeptides with intact GFD of pro-uPA were obtained by limited proteolysis with HLE or V8 protease, while ATF with cleaved GFD was obtained by plasmin treatment.

### *SDS-PAGE and Western blot*

Pro-uPA (10 μM) was incubated with HLE (2.5μM), V8 protease (0.2μM), or plasmin (1μM) in PBS as described above. At indicated intervals, an aliquot was removed and added to

Correspondence: H. Kobayashi, Department of Obstetrics and Gynecology, Hamamatsu University School of Medicine, Handacho 3600, Hamamatsu, Shizuoka, 431-31, Japan.
Received 15 July 1992; and in revised form 12 October 1992.

an aliquot of sample buffer (0.5 M Tris-HCl, pH 6.8, 2.5% SDS, 25% glycerol, with and without 5% 2-mercapto-ethanol), boiling for 3 min, and then immediately analysed by 5–15% sodium dodecyl sulfate-poly acrylamide gel electrophoresis (SDS-PAGE) (Laemmli, 1970). Proteins separated by SDS-PAGE were electroblotted onto polyvinylidine difluoride (PVDF) membrane (Millipore, Bedford, MA), using a semidry electroblotting apparatus (Parmacia-LKB) at 40 mA/gel (90 min, 23°C). After transfer, the sheets were incubated with mouse monoclonal antibody (moAB) 377 (1.0 $\mu g\ ml^{-1}$), reacting with the A-chain of pro-uPA, moAB GFD1 (0.45 $\mu g\ ml^{-1}$), specific for a peptide sequence within Growth Factor-like domain (GFD) of uPA, and moAB 98.6 (0.5 $\mu g\ ml^{-1}$), reacting with the B-chain of pro-uPA in Tris-buffered saline containing 2% BSA, pH 7.5 (TBS-BSA) (1 h, 23°C). The sheets were incubated with biotin-conjugated anti-mouse IgG (0.5 $\mu g\ ml^{-1}$, Sigma) in TBS-BSA (1 h, 23°C), followed by incubation with avidin-peroxidase (4 $\mu g\ ml^{-1}$, Sigma) in TBS-BSA (1 h, 23°C), and developed with 0.1% 4-chloro-1-naphthol, 37% ethanol, 0.005% hydrogen peroxide in TBS.

*Isolation of the ATF-fractions from HLE-, V8 protease-, or plasmin-treated pro-uPA*

Crude ATF-fraction of uPA-molecule was first obtained as a side-fraction by the same method as the preparation of two-chain uPA/LMW-uPA by plasmin (Günzler et al., 1982). The ATF polypeptide formed was separated from two-chain uPA/LMW-uPA by ion-exchange column chromatography. Two mg crude ATF-fraction was subjected to cation-exchange chromatography Mono S HR 5/5 column applying a HPLC-system equilibrated with 50 mM sodium acetate buffer, pH 5.0. After washing with equilibrating buffer, bound proteins were eluted with a linear salt gradient (0.15–0.5 M NaCl) in sodium acetate buffer for 35 min at 23°C. Further purification was achieved by reversed-phase HPLC. Polypeptides were eluted with a linear gradient from 0 to 70% acetonitrile in 0.1% trifluoro-acetic acid/$H_2O$.

*Synthesis of uPA peptides*

uPA20–30 (consisting of amino acid sequences from Val20 to Tyr30 of uPA molecule) and uPA17–34 (from Gly17 to Pro34), representing sequences within the GFD of uPA, were synthesised by the Merrifield-solid-phase method. These synthetic peptides were purified to homogeneity by reversed-phase HPLC.

*N-terminal amino acid sequence determination and amino acid analysis*

The proteinase-mediated cleavage site in pro-uPA was determined by N-terminal amino acid sequence analysis (Schmitt et al., 1989). Separated uPA polypeptides were subjected to six Edman degradation cycles. Sequence analyses were performed with a Beckman 890C spinning cup sequenator.

*Cells and culture*

Ovarian cancer cell line, designated as HOC-I, was established from a recurrent region of ovarian endometrioid carcinoma (Fujii, 1989). The cells were maintained under an atmosphere of 5% $CO_2$ in RPMI 1640 medium supplemented with 10% foetal calf serum (FCS; GIBCO). The addition of purified human plasminogen (100 $\mu g\ ml^{-1}$; Sigma) to HOC-I cells produced significant increases in the specific activity of cell-associated urokinase-type plasminogen activator (uPA), indicating that the HOC-I cells express enzymatically inactive uPA (pro-uPA) on their cellular surfaces, which is cleaved by trace contamination of plasmin in the plasminogen preparation (Stoppelli et al., 1986).

U937 promyeloid cells were grown in RPMI 1640 medium supplemented with 10% FCS. Differentiated U937 cells were obtained by incubation in the above medium containing 1 μM phorbol 12-myristate-13-acetate (PMA). A 3-day incubation period resulted in 60% adherent cells. Adherent cells were detached with PBS containing 1 mM EDTA, harvested by centrifugation (1,200 rpm, 10 min), and used for the binding and competition experiments (Kobayashi et al., 1991).

*Quantitative assessment of binding of uPA polypeptides to the uPA receptor on PMA-stimulated U937 cells by flow cytometry (FCM).*

FCM can substitute for radioisotope binding techniques in order to investigate the structure-function relationship of uPA receptor binding site (Kobayashi et al., 1991). PMA-stimulated U937 cells were treated with 50 mM glycine HCl, 0.5 M NaCl, pH 3.0, first, to dissociate receptor-bound uPA. After centrifugation, the cell pellet was adjusted to a density of $10^6$ cells $ml^{-1}$ in PBS containing 0.1% BSA, pH 7.4 (PBS-BSA). uPA polypeptides derived from pro-uPA by limited proteolysis were used as competitors. 1.5 nM of FITC-pro-uPA in the presence or absence of unlabelled competitors was added to the cells (30 min, 4°C). Nonspecific binding of FITC-pro-uPA to U937 cells was determined in the presence of an excess (0.1 μM) of parent pro-uPA.

*Cell attachment assay*

The fibronectin solution (10 $\mu g\ ml^{-1}$ PBS) was incubated in the 96-well microtiter plate (Costar, Cambridge, MA; 2 h, 23°C) (Ruoslahti et al., 1982). The unattached protein was removed by washing three times with PBS. For the assay, the HOC-I cells were washed three times with PBS-BSA, then 1 mM EDTA was added, and the cells were removed by agitation until they were detached. The cells were then collected by centrifugation, washed with PBS-BSA, and were dispersed by pipetting until a single cell suspension was obtained. Before the last centrifugation, a sample was taken, and cell numbers and viability by trypan blue exclusion were determined. The cells are suspended in PBS-BSA at a concentration of $2 \times 10^6$ cells $ml^{-1}$. One hundred μl of RPMI 1640 containing plasminogen (100 $\mu g\ ml^{-1}$) in the absence or presence of uPA polypeptides, as described in the Figure 9 legend, was added to each well (1 h, 37°C). This was followed by the addition of 100 μl of the cell suspension. After the plate is incubated (2 h, 37°C), the unattached cells are removed simply by tossing out the medium and washing. Under optimal conditions, about 70% of these cells remain attached to the well after washing (Control). The cells are then fixed with 3% paraformaldehyde and stained with 1% toluidine blue, 3% formaldehyde in PBS, and attached cells are counted with an Olympus light microscope ( × 200). The data are expressed as the number of attached cells at randomly chosen area (each sample was assayed using triplicate wells and each well was counted in three areas).

*Cell invasion assay*

Invasiveness of tumour cells was determined using a modification of the Membrane Invasion Culture System (MICS) (Gehlsen et al., 1984; Kleinman et al., 1986; Terranova et al., Albini et al., 1987). Briefly, polycarbonate filters, 8-μm pore size (Costar) were coated with an extract of basement membrane components (Matrigel, 50 μg/filter, 1.2 $\mu g\ mm^{-2}$) and dried (2 h, 37°C). Blind well Boyden chambers were filled with 600 μl RPMI 1640, 0.1% BSA in the lower compartment and coated filters mounted in the chamber. The cells to be studied were collected by short exposure to EDTA resuspended in PBS-BSA, and shaking as described above. The *in vitro* cell invasion assay was carried out to study the role of cell-associated uPA in tumour cell invasion by attempting to saturate uncovered uPA receptors by ATF or enzymatically inactive uPA polypeptides (modifying agents). We added these modifying agents under the conditions described below. The HOC-I cells were pretreated with each modifying agent (at the concentrations noted) as described in the Figure 8 legend, typically for 60 min at 23°C.

Then 100 µl cell suspension ($2 \times 10^6$ ml$^{-1}$) was placed in the upper compartment of the chamber and followed by the addition of 100 µl serum-free medium in the presence of plasminogen (100 µg ml$^{-1}$). Human fibroblast conditioned media were placed in the lower compartment as a source of chemoattractants. Under these conditions, few cells died within 24 h in preliminary experiments. The chemotactic assays were conducted in a similar fashion without coating of Matrigel. After incubation (24 h, 37°C) the filters were then removed, fixed and stained. All material from the upper surface of the filter was carefully removed by scraping with a cotton tip, and invasive cells adhering to the lower surface of the filter were quantitated with a light microscope ( × 200). The data are expressed as the number of cells on the bottom surface of the filter at randomly chosen area (each sample was assayed using triplicate filters and filters were counted in three areas).

**Figure 1** SDS-PAGE and Western blot: specific cleave of pro-uPA by human leucocyte elastase. SDS-PAGE (**a**, 15% acrylamide, nonreducing conditions) of pro-uPA treated with HLE at 37°C for different lengths of time. Lane 1, 0 min; lane 2, 10 min; lane 3, 30 min; lane 4, 1 h; lane 5, 3 h; lane 6, 5 h; lane 7, 8 h; lane 8, 24 h. Western blot with moAB 377 (**b**, reacting with A-chain of uPA) and moAB GFD1 (**c**, reacting with the receptor binding epitope uPA17–34 within the GFD of uPA). Arrow indicates ATF[HLE] with molecular mass of 20 KDa.

### Results

*uPA polypeptides derived from pro-uPA by limited proteolysis of proteinases*

Incubation of pro-uPA with HLE results in the cleavage of uPA at amino acid position 160 and 166, generating enzymatically inactive two-chain uPA (Schmitt *et al.*, 1989). Extensive treatment (96 h, 23°C) of pro-uPA with HLE results in production of two-chain uPA and lower molecular mass uPA fragments (molecular mass = 20 KDa and 14 KDa). Pro-uPA incubated with HLE was analysed by SDS-PAGE and also by Western blot (Figure 1). The 20 KDa and the 14 KDa polypeptides were obtained as a side-fraction by the preparation of two-chain uPA. When the 20 KDa polypeptide were chromatographed by cation-exchange on a Mono S column, one major peptide peak associated with three minor peaks were separated (Figure 2, C-3). A major peptide (designated as ATF[HLE]) further purified by reversed phase HPLC reacts with both moAB 377 and moAB GFD1, while three minor peptides react mainly with moAB GFD1 (by Western blot and Dot blot analysis). The 14 KDa uPA fragment reacts with moAB 98.6, suggesting that this peptide is a degradation product of the B-chain of uPA. Under reducing conditions, the ATF[HLE] showed a single band on SDS-PAGE with a molecular mass of 17 KDa, suggesting that this polypeptide is not a single-chain uPA polypeptide, but consists of the GFD that was not cleaved.

Proteolysis of pro-uPA with V8 protease was visualized by SDS-PAGE and Western blot. The time course digestion was monitored by SDS-PAGE under nonreducing conditions (Figure 3). Virtually complete proteolysis was obtained with 3 h, yielding four major polypeptides with molecular mass of 38 KDa, 33 KDa, 13 KDa and 12 KDa as well as two minor polypeptides with molecular mass of 17 KDa and 5 KDa. The results of Western blot analyses are illustrated in Figure 3. MoAB GFD1 reacts with pro-uPA/two-chain uPA and the polypeptide bands at 38 KDa, 17 KDa and 5 KDa. MoAB GFD1 does not react with the polypeptide bands at 33 KDa, 13 KDa and 12 KDa. MoAB 377 reacts with pro-uPA/two-chain uPA and the polypeptides including 38 KDa, 17 KDa,

**Figure 2** Comparison by Western blot analysis of uPA polypeptides obtained after treatment of pro-uPA by human leucocyte elastase. Various degradation products of pro-uPA were identified by the Western blot technique. **a** and **b**, Coomassie blue stain (SDS-PAGE); **c** and **d**, western blot with moAB 377 (1), moAB 98.6 (2, reacting with B-chain of uPA), and moAB GFDI. (3). **a** and **c**, nonreducing conditions; **b** and **d**, reducing conditions. Arrows indicate two-chain uPA **a**, B-chain, A-chain, ATF[HLE] (**b**, from the top), two-chin uPA (**c**,1), B-chain (**d**,2), and A-chain, ATF[HLE] (**d**,3; from the top).

**Figure 3** SDS-PAGE and Western blot: specific cleavage of pro-uPA by V8 protease. SDS-PAGE (**a**, 15% acrylamide, nonreducing conditions) of pro-uPA treated with V8 protease at 37°C for different lengths of time. Lane 1, 0 min; lane 3, 5 min; lane 4, 40 min; lane 5, 3 h; lane 6, 5 h, lane 7, 8 h, lane 8, 24 h; lane 2, ATF[PL] derived from plasmin-treated pro-uPA. See Figure 5. Western blot with moAB 377 **b** and moAB GFD1 **c**. Arrows indicate ATF[V8] (**a**, lane 4), uPA44–135<52/53>, uPA44–135, uPA4–43 (**a**, lane 8; from the top), ATF[V8], uPA44–135<52/53>, uPA44–135 (**b**, lane 4; from the top), ATF[V8], uPA4–43 (**c**, lane 4; from the top).

**Figure 4** Comparison by dot blot analysis of isolated uPA polypeptides obtained after treatment of pro-uPA by V8 protease. The crude ATF-fraction separated from two-chain uPA by ion-exchange column chromatography was subjected to Mono S column applying a HPLC-system. Further purification was achieved by reversed-phase HPLC. 1, ATF[V8]; 2, uPA4–43; 3, uPA44–135<52/53>; 4, uPA44–135. Dot blot with moAB GFD1 **a** and moAB 377 **b**.

13 KDa, and 12 KDa. Under reducing conditions, the molecular mass of the uPA polypeptide (designated as ATF[V8]) was found to be 17 KDa (data not shown), suggesting that this polypeptide is a single-chain polypeptide consisting of the intact GFD. Regarding the uPA fragment with molecular mass of 13 KDa, under reducing conditions, the molecular mass was found to be 12 KDa due to the removal of the N-terminal amino acid residues Ile44-Glu52. This finding is based on amino acid composition analyses and N-terminal amino acid sequence analyses of the polypeptide (data not shown). Two sequences in an approximately 1:1 ratio were found; the first started with Ile44 (uPA44–52), the second from Gly53 (uPA53–135). Therefore this polypeptide was found to consist of the Kringle that was cleaved between the Glu52 and Gly53 bond (designated as a uPA44–135<52/53>). Regarding the uPA fragment with molecular mass of 12 KDa, under reducing conditions, the molecular mass was found to be the same. This polypeptide was found to consist of the Kringle that was not cleaved (uPA44–135). Also, the uPA fragment with molecular mass of 5 KDa was found to consist of the intact GFD (uPA4–43). MoAB 377 does not react with this polypeptide, while moAB GFD1

**Figure 5** Schematic representation of the pro-uPA molecule and its cleavage sites. Proteolytic cleavage at peptide bond Lys158-Ile159 yields the enzymatically active two-chin uPA-molecule (HMW-uPA) still held together by the disulfide bonds. If pro-uPA, HMW-uPA, and ATF are cleaved within the peptide sequence 20–30, binding to uPA-receptor will not occur. Proteolytic degradations of pro-uPA by other proteinases are described in detail in the reference (Schmitt *et al.*, 1991). Plasmin (P), V8 protease (V), elastase (E).

reacts (Figure 4). The locations of the respective cleavage sites are illustrated in Figure 5. Limited proteolysis of pro-uPA with plasmin at pH 6.0 and subsequent incubation at pH 8.0 (autodigestion) results in the cleavage of HMW-uPA at amino acid position 135, generating LMW-uPA and ATF (designated at ATF[PL] (Stoppelli *et al.*, 1985; Cubellis *et al.*, 1986)) (Figure 6). ATF[PL] contains an additional three polypeptides (see Kobayashi *et al.*, 1992*a*). One represents the intact Kringle (Ser47-Lys135), one ATF with proteolytic cleavage occurring at Lys23-Tyr24 (uPA1- 135<23/24>) and the remaining fragment ATF with proteolytic cleavage, both at Lys23-Tyr24 and at Lys35-Lys36 (uPA1-135<23/24, 35/36>). The amount of intact ATF contained in the crude ATF preparation (ATF[PL]) separated from LMW-uPA is very low (1-2%) (Kobayashi *et al.*, 1992*a*).

### *Binding of uPA polypeptides to the U937 cell receptor assessed by flow cytometry*

Competitive inhibition assays employing flow cytometry (FCM) were performed to examine the affinity of each uPA polypeptide responsible for binding to the uPA receptor on U937 cells. Pro-uPA, HMW-uPA, uPA polypeptides (ATF [HLE], ATF[V8], ATF[PL], uPA4-43, uPA44-135, uPA44-135<52/53>, uPA47-135, uPA1-135<23/24>, and uPA1-135<23/24, 35/36>), and synthetic uPA peptides (uPA20-30 and uPA17-34) were tested for binding to receptors on U937 cells. The results of experiments using PMA-stimulated adherent cells are shown in Figure 7. 50% inhibition of FITC-pro-uPA binding to U937 cells was obtained with 1.5 nM pro-uPA, 3.0 nM HMW-uPA, 12 nM ATF[V8], 20 nM ATF[HLE], 70 nM uPA4-43, 1000 nM uPA20-30, 1000 nM uPA17-34, and > 1,000 nM ATF[PL], respectively. Synthetic uPA peptides have an affinity for the uPA receptor about 667-fold less than pro-uPA as the secondary structure stabilised by the cystein bridges is lost. Also, ATF[PL] has an affinity for the uPA receptor of at least 667-fold less than pro-uPA itself. uPA fragments not containing GFD (uPA44-135, uPA44-135<52/53>, uPA47-135) and ATF with an additional proteolytic cleavage (uPA1-135<23/24>, uPA1-135<23/24,35/36>) do not bind to the receptor.

### *Effect of uPA polypeptides on cancer cell invasion*

If a cell-associated uPA is essential to the invasion process of cancer cells, an inhibition of invasion would be observed after all membrane receptor sites are occupied by enzymatically inactive uPA fragments such as GFD or ATF.

**Figure 6** SDS-PAGE and Western blot: Specific cleavage of pro-uPA by plasmin. SDS-PAGE (**a**, 15% acrylamide, nonreducing conditions) or pro-uPA treated with plasmin at 37°C for different lengths of time. Lane 1, 0 min; lane 2, 10 min; lane 3, 30 min; lane 4 1 h; lane 5, 5 h; lane 6, 24 h; lane 7, 96 h; lane 8, ATF[PL]. Western blot with moAB 377 **b** and moAB GFD1 **c**. Each arrow indicates that LMW-uPA (lane 7), ATF (mixture of uPA1-135<23/24> and uPA1-135<23/24, 35/36>) and Kringle-domain (uPA47-135) (lane 8; from the top).

Under this condition, pro-uPA synthesised and released by tumour cells can not bind to their own receptors, because tumour cells do not have free uPA binding sites. Experiments with uPA polypeptides derived from pro-uPA by limited proteolysis of HLE or V8 protease show a statistically significant inhibition of invasion in a dose-dependent manner in an *in vitro* assay (Figure 8). However, attempts to block invasion with ATF obtained by plasmin-treated pro-uPA (ATF[PL]) showed no significant effects. In addition, syn-

**Figure 7** Inhibition of binding of FITC-conjugated pro-uPA to U937 cell receptors by uPA polypeptides assessed by flow cytometry. Pro-uPA (●), HMW-uPA (○), ATF[V8] (▲), ATF[HLE] (△), uPA20-30 (▼), uPA17-34 (▽), uPA4-43 (■), ATF[PL] (□). Other uPA polypeptides including uPA44-135<52/53>, uPA44-135, uPA47-135, uPA1-135<23/24>, and uPA1-135<23/24, 35/36> have no inhibition (data not shown). SD<10% in all samples.

**Figure 8** Effects of uPA polypeptides on tumour cell invasion. The HOC-I cells were tested in an *in vitro* reconstituted basement membranes invasion assay. The numbers of cells that had attached to the lower surface of the filter at 24 h were used to calculate invasion. One hundred $\mu g\ ml^{-1}$ plasminogen was routinely added to the upper chamber and each cell invasion assay was performed in the presence of plasminogen. Each uPA polypeptide (see Text) and serine proteinase inhibitors (trasylol [$500\ U\ ml^{-1}$] and epsilon-aminocapronic acid [E-ACA; $500\ \mu g\ ml^{-1}$]) were preincubated with HOC-I cells 60 min before the cells were plated on the matrigel. Trasylol and E-ACA reduced the invasion by about 70–80% ($P<0.001$). The concentration used are not cytotoxic. The inhibitory effect of the serine proteinase inhibitors was dose-dependent (see Kobayashi *et al.*, 1992*b*). Control experiments were carried out in the absence of uPA polypeptides with and without plasminogen ($100\ \mu g\ ml^{-1}$). For each experiment three replicates were performed. Mean and SD of three experiments are shown. The similar results were obtained when these agents were added to the wells before the cells were seeded into the culture wells (data not shown here).

thetic uPA peptides, uPA20–30 and uPA17–34, had essentially no effect. To investigate the more complete dose relationship of ATF[HLE] and ATF[V8] on the inhibition of HOC-I cell invasion, different doses of each uPA polypeptide were preincubated with HOC-I cells 60 min before the cells were plated on matrigel. We confirmed that attempts with ATF[V8] and ATF[HLE], but not ATF[PL], showed a significant inhibition of invasion in a dose-dependent manner, and gave more of a sense of reproducibility of this phenomena in replicate experiments (Table I). Also, the data showed that the dose of $10\ \mu g\ ml^{-1}$ used in our experiments achieved near-maximal effects.

The chemotactic response of the cells was also tested to determine whether the inhibition of the HOC-I cells ability to invade was due to the uPA polypeptide inhibiting this response (Figure 9). The cells tested here showed a good chemotactic response in the presence of uPA polypeptides. These results indicate that there are no effects of uPA polypeptides on cancer cell chemotaxis, because the cancer cells are able to migrate to chemoattractants even in the presence of uPA polypeptides.

In addition, the effects of uPA polypeptides used in the experiments on HOC-I cell attachment have been studied. Under optimal conditions, 70% of these cells remain attached to the well after washing. No inhibition of attachment to fibronectin was seen with any of uPA polypeptides. The results are shown in Figure 9.

**Table I** Dose-dependent inhibitory activity of ATF[V8], ATF[HLE] and ATF[PL] on tumour cell invasion

| *Dose of uPA polypeptides ($\mu g\ ml^{-1}$)*[a] | *Cells/Field (mean ± SD)*[b] | | |
|---|---|---|---|
| | *ATF[V8]* | *ATF[HLE]* | *ATF[PL]* |
| 10 | 19.3 ± 3.7[c] | 18.3 ± 2.3[c] | 27.5 ± 4.5 |
| 5 | 18.9 ± 3.5[c] | 19.4 ± 2.9[c] | 31.7 ± 3.8 |
| 2 | 23.0 ± 2.9[d] | 24.0 ± 0.9[d] | 30.6 ± 2.3 |
| 1 | 28.0 ± 2.2[d] | 26.3 ± 3.7 | 32.2 ± 4.0 |
| 0.5 | 30.0 ± 2.1 | 31.2 ± 1.8 | 33.4 ± 3.5 |
| 0.1 | 33.2 ± 2.6 | 30.1 ± 1.9 | 31.0 ± 2.0 |
| 0.01 | 32.4 ± 3.1 | 33.2 ± 1.7 | 32.6 ± 2.5 |
| Control | 31.6 ± 2.4 | 31.6 ± 2.4 | 31.6 ± 2.4 |

[a]uPA polypeptides were preincubated with HOC-I cells 60 min before the cells were plated on the matrigel (Figure 8). Control experiments were carried out in the absence of uPA polypeptides with plasminogen ($100\ \mu g\ ml^{-1}$); [b]For each experiment three replicates were performed. Mean and SD of three experiments are shown. SD < 20% in all samples; [c]$P<0.05$; [d]$P<0.01$.

### Discussion

The central molecule in the surface pathway for plasminogen activation appears to be the uPA receptor (Vassalli, *et al.*, 1985; Blasi *et al.*, 1987; Estreicher *et al.*, 1989). Plasmin, kallikrein, cathepsin B, L, and thermolysin are known to cleave pro-uPA at position 158 producing enzymatically active HMW-uPA, held together by a disulfide bridge (Günzler *et al.*, 1982; Ichinose *et al.*, 1986; Gurewich & Pannell, 1987; Schmitt *et al.*, 1989; Schmitt *et al.*, 1991; Kobayashi *et al.*, 1991; Goretzki *et al.*, 1992). Receptor-bound pro-uPA can be converted to the two-chain form of uPA-molecule, or alternatively can first be activated by plasmin or cathepsin B and then bind to the uPA receptor (Cubellis *et al.*, 1986; Kobayashi *et al.*, 1991). Receptor molecules could be hidden by the enzymatically inactive ATF, after the proeolytic cleavage of receptor-bound uPA at position 135 by plasmin, which might represent a regulatory mechanism sensing the excess of plasmin at the cell surface (Cubellis *et al.*, 1986). Under the condition of a high concentration of plasmin near the cell surface, a part of the pro-uPA produced from the cells could be cleaved in the position of not only the Lys158-ILe159 bond and Lys135-Lys136 bond but also of the Lys23-Tyr24 bond and/or Lys35-Lys36 bond in the GFD before binding to the uPA receptor (Schmitt *et al.*, 1991; Kobayashi *et al.*, 1992*a*). These polypeptides with

**Figure 9** Effects of uPA polypeptides on tumour cell attachment and chemotaxis. □, chemotactic response (Cells/Field; %); ■, cell attachment (Attached cells/Field; %). SD<20% in all samples.
*Chemotactic assay:* The chemotactic assay was conducted in a similar fashion without coating of matrigel. The number of cells on the bottom surface of the filter in the absence of uPA polyeptides were designated at 100%. *Cell attachment assay:* HOC-I cells were seeded on fibronectin in the presence and absence of uPA polyeptides. After 2 h later, 70% of the cells attached to the wells in the absence of uPA polypeptides (Control). These values were designated at 100%. Each experiment was performed in the presence of plasminogen (100 μg $ml^{-1}$).

the cleavage occurring in the GFD can not bind to uPA receptor on U937 cells any more, providing an additional feedback mechanism of regulation of uPA binding to the receptor (Kobayashi *et al.*, 1992*a*).

Our preliminary study indicates that (1) ovarian cancer derived HOC-I cells used in this study have uPA molecules on their cell surfaces and most of the cell-associated uPA on the cell surfaces are enzymatically inactive pro-uPA, that (2) pro-uPA is bound to a specific surface receptor that is incompletely saturated, that (3) cell-associated pro-uPA can be converted to HMW-uPA by plasmin and cathepsin B, and that (4) its activity can be inhibited by an antibody against B-chain of uPA (Kobayashi *et al.*, 1992*b*). We attempted to isolate uPA polypeptides with intact GFD from proteinase-treated pro-uPA to determine whether uPA polypeptides can inhibit HOC-I cell invasion in an *in vitro* assay, when all membrane receptor sites are saturated by enzymatically inactive uPA fragments such as intact GFD or ATF. The significance of the expression of cell surface uPA activity on invasive potential was examined by saturation of free uPA receptor with enzymatically inactive uPA polypeptides. uPA polypeptides derived from pro-uPA by limited proteolysis of HLE or V8 protease significantly inhibit invasion in a dose-dependent manner. This inhibition may correlate with the quantities of uPA polypeptides available to saturate uPA receptors. Furthermore, we conclude that the mode of action of this peptide is not due to inhibition of cancer cell attachment and cell chemotaxis. The above mentioned uPA polypeptides appeared capable of blocking invasion not only when they were added with the cells but also when they were preadsorbed on to the basement membrane matrigel (data not shown). On the contrary ATF obtained from plasmin-treated uPA (ATF[PL]) did not affect the invasive potential of the cells. Also, synthetic uPA peptides had essentially no effect, indicating that both an intact peptide sequence 20–30 and the conformation of GFD are prerequisites for binding of uPA-molecules (Schmitt *et al.*, 1991) and for inhibition of tumour cell invasion.

Various investigators (Liotta *et al.*, 1980; Thorgeirsson *et al.*, 1982; Ostrowski *et al.*, 1986; Bergman *et al.*, 1986; Nakajima *et al.*, 1987; Reich *et al.*, 1988; Hearing *et al.*, 1988; Cajot *et al.*, 1989; Baker *et al.*, 1990; Ellis *et al.*, 1990) have shown that tumour cells may express surface-associated uPA activity and that inhibition of this activity by specific antibodies or proteinase inhibitors leads to a decrease in the invasive potential of tumour cells.

The use of antibodies in therapy of humans is highly restricted. Our data indicate that enzymatically inactive receptor-bound uPA polypeptides can also lead to a decrease in the invasive potential of this tumour cell line and would be worth testing *in vivo*. The uPA receptor on HOC-I cells would be hidden by the enzymatically inactive uPA polypeptides, which leads to a decrease in a proteolytic action on tumour cell surfaces.

The incompleteness with which the competitive receptor-bound uPA polypeptides block invasion indicates that uPA is not sufficient in invasion mechanisms, suggesting that HOC-I cells have a significant uPA/plasmin-independent proteinase activity (Kobayashi *et al.*, 1992*b*). We did not examine additional evidence for the participation of other proteinases such as metalloproteinases. Other explanations are that (1) pro-uPA/uPA-bound receptors are not reoccupied by exogenous uPA fragments/peptides, that (2) binding sites of receptor-bound uPA polypeptides, which were added exogenously, may be cleaved by trace contamination of plasmin in the plasminogen preparation, and that (3) the affinity of uPA polypeptides to uPA receptors is lower than that of pro-uPA/HMW-uPA produced by cancer cells, suggesting that uPA receptors may be reoccupied by uPA-molecules released from tumour cells. Notwithstanding these limitations, the present study strongly argues for a role of the cell-associated uPA/receptor-system in facilitating the *in vitro* invasion of ovarian cancer cells.

The authors wish to acknowledge the extremely helpful critiques of Dr N. Kanayama (Hamamatsu University School of Medicine).

### References

ALBINI, A., IWAMOTO, Y., KLEINMAN, H.K., MARTIN, G.R., AARONSON, S.A., KOZLOWSKI, J.M. & MCEWAN, R.N. (1987). A rapid *in vitro* assay for quantitating the invasive potential of tumor cells. *Cancer Res.*, **47**, 3239–3245.

APPELLA, E., ROBINSON, E.A., ULLRICH, S.J., STOPPELLI, M.P., CORTI, A., CASSANNI, G. & BLASI, F. (1987). The receptor-binding sequence of urokinase. *J. Biol. Chem.*, **262**, 4437–4440.

BAKER, M.S., BLEAKLEY, P., WOODROW, G.C. & DOW, W.F. (1990). Inhibition of cancer cell urokinase plasminogen activator by its specific inhibitor PAI-2 and subsequent effects on extra-cellular matrix degradation. *Cancer Res.*, **50**, 4676–4684.

BERGMAN, B.L., SCOTT, R.W., BAJPAI, A., WATTS, S. & BAKER, J.B. (1986). Inhibition of tumour cell-mediated extracellular matrix degradation by a fibroblast proteinase inhibitor, protease nexin I. *Proc. Natl Acad. Sci. USA*, **83**, 996–1000.

BLASI, F., VASSALLI, J.-D. & DANO, K. (1987). Urokinase-type plasminogen activator: proenzyme, receptor and inhibitors. *J. Cell Biol.*, **104**, 801–804.

CAJOT, J.F., SCHLEUNING, W.D., MEDCALF, R.L., BAMAT, J., TESTUZ, J. LIEBERMANN, L. & SORDAT, B. (1989). Mouse L cells expressing human prourokinase-type plasminogen activator: effects on extra-cellular matrix degradation and invasion. *J. Cell Biol.*, **109**, 915–925.

CUBELLIS, M.V., NOLLI, M.L., CASSANI, G. & BLASI, F. (1986). Binding of single-chain prourokinase to the urokinase receptor of human U937 cells. *J. Biol. Chem.*, **261**, 15819–15822.

DANO, K., ANDREASEN, P.A., GRONDAHL-HANSEN, J., KRISTENSEN, P.I., NIELSEN, L.S. & SKRIVER, L. (1985). Plasminogen activators, tissue degradation, and cancer. *Adv. Cancer Res.*, **44**, 139–266.

DELROSSO, M., DINI, G. & FIBBI, G. (1985). Receptors for plasminogen activator, urokinase, in normal and Rous sarcoma virus-transformed mouse fibroblasts. *Cancer Res.*, **45**, 630–636.

ELLIS, V., WUN, T.-C., BEHRENDT, N., RONNE, E. & DANO, K. (1990). Inhibition of receptor-bound urokinase by plasminogen activator inhibitors. *J. Biol. Chem.*, **265**, 9904–9908.

ESTREICHER, A., WOHLWEND, A., BELIN, D., SCHLEUNING, W.-D. & VASSALLI, J.D. (1989). Characterization of the cellular binding site for the urokinase-type plasminogen activator. *J. Biol. Chem.*, **264**, 1180–1189.

FUJII, T. (1989). Establishment and characterization of human ovarian endometrioid carcinoma cell line. *Acta. Obstet. Gynaec. Jpn.*, **41**, 161–166.

GEHLSEN, F.R., WAGNER, H.N. & HENDRIX, M.J.C. (1984). Membrane Invasion Culture System (MICS). *Med. Instrum.*, **18**, 268–271.

GORETZKI, L., SCHMITT, M., MANN, K., CALVETE, J., CHUCHOLOWSKI, N., KRAMER, M., GÜNZLER, W.A., JÄNICKE, F. & GRAEFF, H. (1992). Effective activation of the proenzyme form of the urokinase-type plasminogen activator (pro-uPA) by the cysteine protinase cathepsin L. *FEBS Lett.*, **297**, 112–118.

GÜNZLER, W.A., STEFFENS, G.J., ÖTTING, F., BUSE, G. & FLOHE, L. (1982). The primary structure of high molecular mass urokinase from human urine. The complete amino acid sequence of the A-chain. *Hoppe Seyler's Z. Physiol. Chem*, **363**, 1155–1165.

GUREWICH, V. & PANNELL, R. (1987). Inactivation of single-chain urokinase (pro-urokinase) by thrombin and thrombin-like enzymes: Relevence of the findings to the interpretation of fibrin-binding experiments. *Blood*, **69**, 769–772.

HEARING, V.J., LAW, L.W., CORTI, A., APPELLA, E. & BLASI, F. (1988). Modulation of metastatic potential by cell surface urokinase of murine melanoma cells. *Cancer Res.*, **48**, 1270–1278.

ICHINOSE, A., FUJIKAWA, K. & SUYAMA, T. (1986). The activation of pro-urokinase by plasma kallikrein and its inactivation by thrombin. *J. Biol. Chem.*, **261**, 3486–3489.

KLEINMAN, H.K., MCGARVEY, M.L., HASSELL, J.R., STAR, V.L., CANNON, F.B., LAURIE, G.W. & MARTIN, G.R. (1986). Basement membrane complexes with biological activity. *Biochemistry*, **25**, 312–318.

KOBAYASHI, H., SCHMITT, M., GORETZKI, L., CHUCHOLOWSKI, N., CALVETE, J., KRAMER, M., GÜNZLER, W.A., JÄNICKE, F. & GRAEFF, H. (1991). Cathepsin B efficiently activates the soluble and the tumor cell receptor-bound form of the proenzyme urokinase-type plasminogen activator (pro-uPA). *J. Biol. Chem.*, **266**, 5147–5152.

KOBAYASHI, H., OHI, H., MONIWA, N., SHINOHARA, H., MAEDA, M. & TERAO, T. (1992*a*). Regulation of plasmin production by cancer cells. *Jpn. J. Obstet. Gynecol. Neonatal. Hematol.*, **12**, 39–48.

KOBAYASHI, H., OHI, H., SUGIMURA, M., SHINOHARA, H., FUJII, T. & TERAO, T. (1992*b*). Inhibition of *in vitro* ovarian cancer cell invasion by modulation of urokinase-type plasminogen activator and cathepsin B. *Cancer Res.*, **52**, 3610–3614.

LAEMMLI, U.K. (1970). Cleavage of structural proteins during the assembly of the head of bacteriophage T4. *Nature*, **227**, 680–685.

LIOTTA, L.A., TRYGGVASON, K., GARBISA, S., HART, I., FOLTZ, C.M. & SHAFIE, S. (1980). Metastatic potential correlates with enzymatic degradation of basement membrane collagen. *Nature*, **284**, 67–68.

LIOTTA, L.A., RAO, C.N. & BARSKY, S.H. (1983). Tumor invasion and the extracellular matrix. *Lab. Invest.*, **49**, 636–649.

MIGNATTI, P., ROBBINS, E. & RIFKIN, D.B. (1986). Tumor invasion through the human amniotic membrane: requirement for a proteinase cascade. *Cell*, **47**, 489–498.

NAKAJIMA, M., WELCH, D.R., BELLONI, P.N. & NICHOLSON, G.L. (1987). Degradation of basement membrane type IV collagen and lung subendothelial matrix by rat mammary adenocarcinoma cell clones of differing metastatic potentials. *Cancer Res.*, **47**, 4846–4876.

OSSOWSKI, L. (1988). *In vivo* invasion of modified chorioallantoic membrane by tumor cells: the role of cell surface-bound urokinase. *J. Cell Biol.*, **107**, 2437–2445.

OSSOWSKI, L. & REICH, E. (1980). Experimental model for quantitative study of metastasis. *Cancer Res.*, **40**, 2300–2309.

OSSOWSKI, L. & REICH, E. (1983). Antibodies to plasminogen activator inhibit human tumor metastasis. *Cell*, **35**, 611–619.

OSTROWSKI, L.E., AHSAN, A., SUTHAR, B.P., PAGAST, P., BAIN, D.L., WONG, C., PATAL, A. & SCHULTZ, R.M. (1986). Selective inhibition of proteolytic enzymes in an in vitro mouse model for experimental metastasis. *Cancer Res.*, **46**, 4121–4128.

PICONE, R., KAJTANIAK, E.L., NIELSEN, L.S., BEHRENDT, N., MASTRONICOLA, M.R., CUBELLIS, M.V., STOPPELLI, M.P., PEDERSEN, S., DANO, K. & BLASI, F. (1989). Regulation of urokinase receptors in monocytelike U937 cells by phorbol ester phorbol myristate acetate. *J. Cell Biol.*, **108**, 693–702.

REICH, R., THOMPSON, E.W., IWAMOTO, Y., MARTIN, G.R., DEASON, J.R., FULLER, G.C. & MISKIN, R. (1988). Effects of inhibitors of plasminogen activator, serine proteases, and collagenase IV on the invasion of basement membranes by metastatic cells. *Cancer Res.*, **48**, 3307–3312.

RUOSLAHTI, E., HAYMAN, E.G., PIERSCHBACHER, M. & ENGVALL, E. (1982). Fibronectin: purification, immunochemical properties, and biological activities. *Method in Enzymol.*, **82**, 828–831.

SCHMITT, M., KANAYAMA, N., HENSCHEN, A., HOLLRIEDER, A., HAFTER, R., GULBA, D., JÄNICKE, F. & GRAEFF, H. (1989). Elastase released from human granulocytes stimulated with N-formyl-chemotactic peptide prevents activation of tumor cell pro-urokinase (pro-uPA). *FEBS Lett.*, **255**, 83–88.

SCHMITT, M., GORETZKI, L., JÄNICKE, F., CALVETE, J., EULITZ, M., KOBAYASHI, H., CHUCHOLOWSKI, N. & GRAEFF, H. (1991). Biological and clinical relevance of the urokinase-type plasminogen activator (uPA) in breast cancer. *Biomed. Biochem. Acta.*, **50**, 731–741.

STEPHENS, R.W., PÖLLÄNEN, J., TAPIOVAARA, H., LEUNG, K.-C., SIM, P.-S., SALONEN, E.-M., RONNE, E., BEHRENDT, N., DANO, K. & VAHERI, A. (1989). Activation of pro-urokinase and plasminogen on human sarcoma cells: A proteolytic system with surface-bound reactants. *J. Cell. Biol.*, **108**, 1987–1995.

STOPPELLI, M.P., CORTI, A., SOFFIENTINI, A., CASSANI, G., BLASI, F. & ASSOIAN, R.K. (1985). Differentiation-enhanced binding of the amino-terminal fragment of human urokinase plasminogen activator to a specific receptor on U937 monocytes. *Proc. Natl Acad. Sci. USA*, **82**, 4939–4943.

STOPPELLI, M.P., TACCHETTI, C., CUBELLIS, M.V., CORTI, A., HEARING, V.J., CASSANI, G., APPELLA, E. & BLASI, F. (1986). Autocrine saturation of pro-urokinase receptors on human A431 cells. *Cell*, **45**, 675–684.

TERRANOVA, V.P., HUJANEN, E.S. & MARTIN, G.R. (1986). Basement membrane and the invasive activity of metastatic tumor cells. *J. Natl Cancer Inst.*, **77**, 311–316.

THORGEIRSSON, U.P., LIOTTA, L.A., KALEBIC, T., MARGULIES, I.M., THOMAS, K., RIOS-CANDELORE, M. & RUSSO, R.G. (1982). Effect of natural protease inhibitors and a chemoattractant on tumor cell invasion *in vitro*. *J. Natl Cancer Inst.*, **69**, 1049–1054.

VASSALLI, J.-D., BACCINO, D. & BELIN, D. (1985). A cellular binding site for the Mr 55,000 form of the human plasminogen activator, urokinase. *J. Cell. Biol.*, **100**, 86–92.